# Genetic Diversity, Conservation, and Utilization of Plant Genetic Resources

**DOI:** 10.3390/genes14010174

**Published:** 2023-01-09

**Authors:** Romesh Kumar Salgotra, Bhagirath Singh Chauhan

**Affiliations:** 1 School of Biotechnology, Sher-e-Kashmir University of Agricultural Sciences and Technology of Jammu, Chatha, Jammu 180009, India; 2 Queensland Alliance for Agriculture and Food Innovation (QAAFI), The University of Queensland, Gatton, QLD 4343, Australia

**Keywords:** genetic diversity, in situ conservation, ex situ conservation, crop improvement, plant genetic resources

## Abstract

Plant genetic resources (PGRs) are the total hereditary material, which includes all the alleles of various genes, present in a crop species and its wild relatives. They are a major resource that humans depend on to increase farming resilience and profit. Hence, the demand for genetic resources will increase as the world population increases. There is a need to conserve and maintain the genetic diversity of these valuable resources for sustainable food security. Due to environmental changes and genetic erosion, some valuable genetic resources have already become extinct. The landraces, wild relatives, wild species, genetic stock, advanced breeding material, and modern varieties are some of the important plant genetic resources. These diverse resources have contributed to maintaining sustainable biodiversity. New crop varieties with desirable traits have been developed using these resources. Novel genes/alleles linked to the trait of interest are transferred into the commercially cultivated varieties using biotechnological tools. Diversity should be maintained as a genetic resource for the sustainable development of new crop varieties. Additionally, advances in biotechnological tools, such as next-generation sequencing, molecular markers, in vitro culture technology, cryopreservation, and gene banks, help in the precise characterization and conservation of rare and endangered species. Genomic tools help in the identification of quantitative trait loci (QTLs) and novel genes in plants that can be transferred through marker-assisted selection and marker-assisted backcrossing breeding approaches. This article focuses on the recent development in maintaining the diversity of genetic resources, their conservation, and their sustainable utilization to secure global food security.

## 1. Introduction

Genetic diversity is the amount of genetic variability present among individuals of a variety or a population within a species. It is the product of the recombination of genetic material (DNA) during the inheritance process, mutations, gene flow, and genetic drift [1], and it results in variations in DNA sequence, epigenetic profiles, protein structure or isoenzymes, physiological properties, and morphological properties. The diversity among plant and animal populations is determined by the hereditary material present in the reproducing members of the population. Genetic diversity is the main driving force for the selection and evolution of populations. Within crop species, the selection of individuals can be natural or artificial, depending upon the variation present [2]. Genetic diversity can be distilled down to the alleles of a gene present in the population, their effects, and their distribution. Genetic diversity is crucial for a healthy population as it maintains different genes that could lead to resistance to pests, diseases, or other stress conditions. It also enables individuals to adapt to various biotic and abiotic stresses. Under environmental changes, different crop varieties survive due to the presence of genetic variation, which enables the varieties to adapt. However, the varieties with little or no genetic diversity could become susceptible to biotic and abiotic stresses. Genetic diversity helps breeders to maintain the crossbred varieties, which leads to sustaining the desirable traits of the varieties, such as quality characteristics and tolerance to various stresses.

In general, plant genetic resources (PGRs) are the total hereditary material, which includes all the alleles of various genes, present in a crop species, including horticulture and medicinal plants, and their wild relatives. They can also be defined as any type of reproductive or vegetative propagating material of the plant species. PGRs include newly developed varieties, cultivated crop varieties, landraces, modern cultivars, obsolete cultivars, breeding stocks, wild forms, weedy forms, wild species of cultivated crops, and genetic stocks, including current breeders’ lines, elite lines, and mutants. These are the building blocks for the genetic improvement of agricultural and industrial crops [3]. Most of the agro-industry and agro-processing sectors also rely on PGRs. These are the pillars of crop development programs, and world food security depends upon the extent of genetic diversity present in PGRs [4]. PGRs are used in crop improvement programs, particularly in the varietal developmental programs. These resources are also used in systematic studies, such as evolutionary biology, cytogenetic, biochemical, physiology, phylogenetic, ecological research, pathology, molecular studies, etc. PGRs encompass all cultivated, wild relatives of cultivated species, traditional cultivars, landraces, and advanced breeding lines of plants [5]. The demand for these PGRs will increase in the future to feed the ever-increasing global population. Moreover, the precedential increase in the world population has resulted in the over-exploitation of PGRs, which has led to the genetic erosion of important germplasm from habitats. Food security issues are of global significance, and genetic resources are being lost at alarming rates due to anthropogenic effects such as genetic erosion, over-exploitation of PGRd, population growth, and climate change. Moreover, with the development and introduction of high-yielding crop varieties, genetic diversity among plant genotypes is declining [6]. The situation is further exacerbated by the frequent recurrence of biotic and abiotic stresses, resulting in a huge loss of PGRs. To avoid this catastrophic situation, there is a need to protect these valuable resources from genetic erosion and use them judiciously. To meet the current, as well as future, global challenges, PGRs need to be explored, collected, conserved, and utilized sustainably. Additionally, the survey, exploration, collection, preservation, and sustainable utilization of PGRs in an organized way is the responsibility of all nations. Researchers, policymakers, and planners have already begun to plan for the proper conservation and sustainable utilization of PGRs for the benefit of society [7].

Maintaining diversity in PGRs is vital for the development and genetic improvement of crop varieties. Presently, the plant species extinction rate is skyrocketing, and life on Earth is facing a sixth mass extinction event caused by climate change and anthropogenic activities, which may lead to ecological collapse [8]. The Leipzig Declaration [9] emphasized saving the seed and planting material to avoid genetic vulnerability and shortage of food under adverse conditions. The diverse gene pool of plant species, such as wild species, landraces, breeding stock, etc., could hold the tools for survival and adaptation under adverse climatic conditions [10]. The conservation and sustainable utilization of these valuable resources is crucial to ensure food security for future generations. Genetic resources should be easily available to plant breeders for the continuous development of new crop varieties. PGRs are an important reservoir of disease- and insect/pest-resistant genes, through which improved and immune crop varieties can be developed. In the present scenario of climate change, PGRs have played a significant role in the development of climate-resilient crop varieties to strengthen food security [11]. By using PGRs, crop varieties are being developed with better yield and quality traits along with resistance to biotic and abiotic stresses, such as diseases, insect pests, flooding, salinity, and drought. Additionally, developing countries rely on PGRs to create more diverse crops. The need for PGRs has risen continually for developing varieties of different crops such as cereals, pulses, vegetables, fruits, and ornamentals [11].

Today, the conservation and sustainable use of PGRs is a priority of the global community to solve issues surrounding food security and other problems arising from increased population growth. In the future, these resources will completely vanish if proper and stringent PGR conservation practices and policies are not implemented [11]. This challenge can be overcome by bringing all stakeholders, including farmers, ethnobotanists, indigenous knowledge-holding people, plant breeders, NGOs, seed banks, and policymakers together to share information, create PGR diversity awareness, develop new technologies, and deploy systematic and scientific conservation. Biotechnological techniques, such as cryopreservation, molecular markers, high-throughput sequencing, and genetic engineering, have improved the conservation of endangered and rare PGRs. Priority should also be given to the exploration of local germplasm and underutilized crop species and the maximum utilization of traditionally local landraces, with the involvement of local people. The Convention on Biological Diversity (CBD) and international undertaking on PGRs [12] are working in harmony for the conservation and sustainable utilization of PGRs under the umbrella of the Earth Summit of the United Nations Conference on Environment and Development (UNCED). A well-planned strategic and forward-looking vision is required for the conservation and sustainable utilization of these genetic resources.

## 2. Importance of Genetic Diversity in Plant Genetic Resources

**Figure 1:**
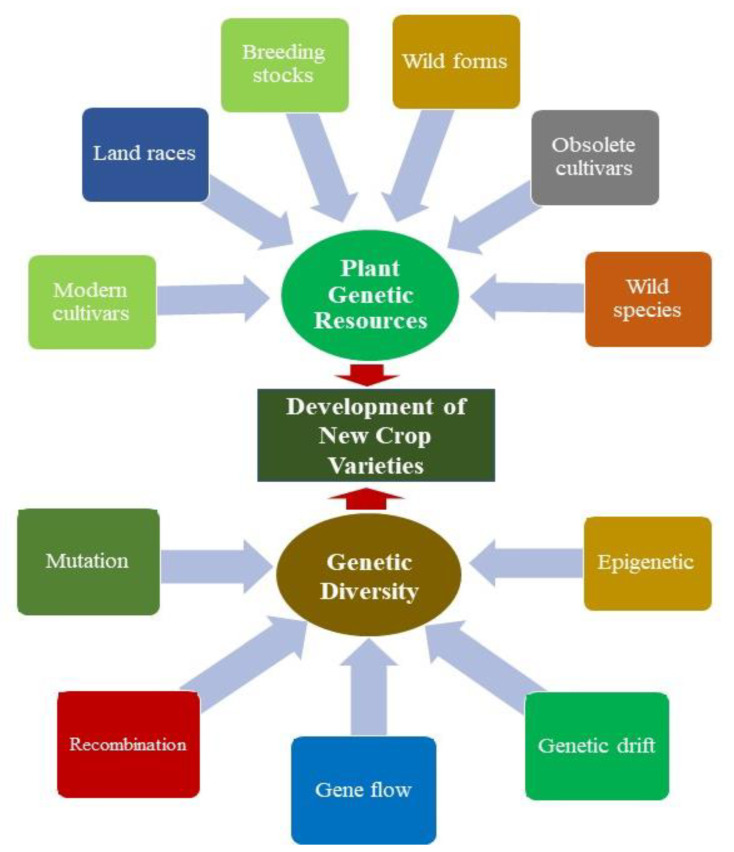
Different sources of genetic diversity and their potential utilization in the development of new crop varieties.

Genetic diversity is the genetic base for crop improvement [13]. Diverse PGRs enable plant breeders to develop or improve crop varieties with desirable qualities. While developing new cultivars, due consideration must be given to the farmers’ preferences, such as high-yielding varieties, quality, and resistance to diseases and insect pests. In ancient times, humans selected desirable genotypes based on natural genetic variability in the population [14]. The preference for the development of new crop varieties shifts over a period with environmental changes. Plant breeders develop climate-resilient varieties possessing all the desirable traits, including resistance to various biotic and abiotic stresses. Genetic diversity in the form of mutant lines, wild species, breeding stocks, etc., is used for the improvement and development of modern crop varieties [13]. For the development of climate-resilient varieties, novel genes tolerant to biotic and abiotic stresses need to be conserved for future use in breeding programs. Additionally, the plant genotypes possessing genes for quality traits and aesthetic properties should be preserved in the available germplasm. Genetic diversity within and between plant species allows plant breeders to select superior genotypes, which can then be used for the development of genetic stock for hybridization programs or the release of a crop variety [13]. Genetic diversity enables PGRs to adapt to varied climatic conditions [14,15]. Moreover, the negative impact of inbreeding in populations can be reduced by enhancing genetic diversity. Higher levels of genetic diversity in PGRs support resilience to adverse environmental changes, integrity, community structure, and ecosystem functions [16,17]. Additionally, it helps plant breeders to utilize genetically diverse parents in a breeding program to improve the productivity of varieties of agriculture and horticulture crops [18] (Figure 1).

Genetic diversity in plant species depends on the heritable variation present within and between populations. It occurs due to genetic variation in the nucleotide sequence of DNA, chromosome mutations, and recombination during sexual reproduction [19]. In the sexual reproduction of plant species, the F_1_ and advanced generations are developed by crossing two or more diverse parents. The offspring developed from two genetically diverse parents possess genetic variations because of recombination during meiosis. Hence, genetically dissimilar offspring from parents are produced. However, this is the genetic material of individuals underlying the variability within, as well as between, species [20]. Generally, genetic diversity can be observed at three levels: diversity between species, diversity between populations within one species, and diversity between individuals within one population. It is genetic variability that provides evolutionary flexibility, resilience, and adaptability in plant species [21]. Before the identification of diverse parents in plant breeding programs, breeders and biotechnologists used a multitude of techniques for the characterization of the germplasm to know the genetic diversity [22]. For the characterization of the genetic diversity of PGRs and the identification of superior genotypes, various techniques are used, such as phenotypic or morphological traits, biochemical or allozyme techniques, and molecular techniques.

## 3. Factors Affecting Genetic Diversity

Genetic diversity changes over time owing to several factors. The main factors responsible for changes in genetic diversity are mutation, selection, genetic drift, and gene flow. Over time, natural and artificial selections play a substantial role in the choosing of superior genotypes, which significantly affects the gene and genotypic frequencies of the population [23]. As per Charles Darwin’s theory of evolution (1859), the desired genotypes are selected for and passed onto subsequent generations [24,25,26]. However, the domestication of desirable genotypes results from the superior genotypes being selected by farmers and breeders and neglects other undesirable genotypes. This leads to a reduction in inferior alleles over generations. During evolution, various morphological, physiological, and biochemical changes take place in plant species and can take different directions under domestication depending on the part of the plant used. Some plant species lose their sexual reproduction during selection for large size of the tuber or root, which is associated with selection for polyploid types, resulting in sterility. Some polyploid plant species, such as allohexaploid wheat and potato, show diploidization behavior during sexual reproduction. Some crops have been turned into annuals from their original form of perennials. In the domestication process, the complete genetic transformation of wild species occurs in the development of modern cultivars through natural and artificial selection [23]. After some time, some domesticated cultivars become susceptible to diseases and pests, which can be improved by incorporating genes from wild plant relatives [27]. During the process of domestication, desirable traits have been selected by breeders as per their preferences [27,28]. However, plant breeders prefer to choose crop varieties with a high yield, resistance to biotic and abiotic stresses, wide adaptation, non-shattering nature, large-sized seeds, early maturing, good quality traits, etc. [29,30]. The main factors affecting genetic diversity will be addressed in the following subsections.

### 3.1. Mutation

Mutations are sudden heritable changes that occur due to aberrations in the nucleotide sequence of DNA. A mutation is the source of genetic variation impacting the phenotype in crop species. Genetic diversity caused by mutations can have neutral, positive, and negative impacts on various characteristics of a plant species. Genetic variations caused by mutations in DNA are the principal cause of changes in the allele frequencies in a population besides selection and genetic drift. From the beginning, natural or spontaneous mutations have played a significant role in creating the genetic variation that has led to food security [31]. Mutations are the ultimate source of plant evolution when they frequently encounter environmental changes. Mutation rates proceed rapidly in response to environmental changes or even changes in the demographical locations related to the socio-economic conditions of the human population in a geographical area. Stress-inducible mutagenesis has been observed because of the use of different external inputs which accelerate adaptive evolution in plants. During mutagenesis, many kinds of genetic changes have been observed such as insertions, deletions, copy number variations, gross chromosomal rearrangements, and the movement of mobile elements. Earlier plant breeders utilized natural mutations as the main source of genetic variation for improving and developing crop varieties. However, modern technologies have accelerated the process by inducing mutation through mutagenesis The concept of mutation breeding was introduced to create more genetic diversity among crop species to improve traits such as disease and insect pest resistance, tolerance to abiotic stresses, and nutritional enhancement in crop varieties [32].

### 3.2. Selection

Natural and artificial selections act on the phenotypic characteristics of the plant species. The phenotypic expression of the plant species depends upon the heritable and non-heritable components in which the genotype–environment interaction also plays a significant role. The selection of superior genotypes depends on the availability of genetic variation present in the plant species. Artificial selection is effective only when sufficient genetic variation is present in the population. The genetic improvement of a genotype depends on the magnitude of genetic variability present in the population, as well as the nature of the association between different components. For example, the level of association of yield traits with other characteristics of the plant species enables the selection of various traits at a time [33]. Plant breeders make effective selection depending on the presence of substantial genetic variation in the population to enhance the maximum genetic yield potential of crop varieties [34]. It also helps in selecting better parents to be used in hybridization programs. Hence, the effective selection of genotypes in a population also depends on the degree of genetic variation in the population.

### 3.3. Migration

Migration is the movement of alleles from one species to another or from one population to another. It occurs through the movement of pollen and seed dispersal and planting material such as rhizomes, suckers, and other vegetative propagating materials. The rate of migration is affected by reproduction cycles and the dispersion of seeds and pollens. Migration can also occur through the moving or shifting of the germplasm from one area to another, which results in the mixing of two or more alleles through pollen and seeds [35].

### 3.4. Genetic Drift

Genetic drift is a mechanism in which the gene and allele frequencies of a population change due to sampling errors over generations. The sampling error changes the allele frequencies by chance, which ultimately changes the genetic diversity over generations. Every pollen grain has a different combination of alleles and can be carried by insects, wind, humans, or other means for hybridization with compatible flowers, largely determined by chance. Thus, in every reproduction cycle, the genetic diversity in crop species is lost at every generation through these chance events [36].

## 4. Factors That Cause Genetic Vulnerability

Over the past century, it has been observed that the genetic diversity in wild populations is declining globally [16,37]. Genetically distinct populations for most species are also declining due to the shrinkage of geographic ranges and lack of proper management and conservation practices [38,39,40]. Most genetic diversity is lost due to infrastructure development, climate change, habitat fragmentation, population reduction, overgrazing, and overharvesting [41]. Besides this, the following subsections describe the major components responsible for the genetic vulnerability of genetic resources.

### 4.1. Narrow Genetic Base of Crop Varieties

The main reason for genetic erosion and vulnerability is the cultivation of genetically uniform cultivars with a narrow genetic base. Indigenous or traditional crop varieties have a broad genetic base, and these cultivars can tolerate various biotic and abiotic stresses [42]. Traditional crop varieties have a low genotype–environment interaction, enabling the genotypes to withstand an epidemic of disease, insect pest incidence, and other adverse environmental conditions [43]. Moreover, pathogen races are less prone to infesting traditional varieties because of the broad genetic base of these varieties compared to the modern released varieties that have common parents. Hybrids have been developed by crossing different genetically uniform inbred lines, which significantly decreased genetic diversity. Additionally, most high-yielding crop varieties have been developed by crossing common parents possessing similar genetic backgrounds, which can significantly reduce the genetic bases of the varieties [22,44].

### 4.2. Wide Spread of Dominant Varieties

The widespread cultivation of a single crop variety over a large area causes genetic vulnerability. These varieties may perform well for a short period but may become susceptible to several diseases and pests. Vertical resistance occurs due to the presence of oligogenic or monogenic resistance, and horizontal resistance occurs due to the presence of polygenes. Sometimes the vertical resistance present in the modern cultivar may show resistance against a disease, but will become susceptible if the pathogen evolves. In vertical resistance, the race of the pathogen or the insect pest biotype interacts with the host and overcomes the monogenic resistance present in the modern cultivar, and the variety becomes susceptible to a particular pathogen or biotype.

### 4.3. Unplanned Introduction of New Plant Species

Sometimes, new high-yielding plant species are introduced and used in a breeding program without proper screening for disease and insect pest resistance, which may result in an unpredicted epidemic of diseases. An example of this is the unplanned introduction of the Texas male sterile (TMS) genotype of maize for the development of hybrid maize genotypes in the USA in 1970. Newly developed maize hybrids had all the desirable characteristics and resistance to most of the common maize diseases. The TMS hybrids were widely cultivated in the USA, covering more than 90% of the maize area. However, these hybrids were susceptible to fungal strains and southern corn leaf blight (*Helminthosporium maydis*). The southern corn leaf blight disease colonized and spread widely, and the whole maize crop was wiped out. If the TMS, a source of male sterility, had been tested and screened properly before use in hybrid breeding programs, or if the monoculture of TMS hybrids had been avoided, the spread of this epidemic could have been countered [45].

## 5. Conservation of Plant Genetic Resources

Since the beginning of agriculture, for a time, the selection, cultivation, and conservation of seeds of locally acclimated plants, also known as called “landraces”, were practiced [46]. This process continued until the rediscovery of Gregor Mendel’s work in the 20th century. This work led to the introduction of breeding programs for the development of high-yielding and stress (biotic and abiotic)-tolerant crop varieties. In the middle of the last century, it laid the foundation for the “Green Revolution” and brought about an exponential increase in agricultural production. However, this led to the replacement of landraces and the expansion of the monoculture cropping system. Over 75% of the genetic diversity in PGRs and 90% of the crop varieties were lost and disappeared from farmers’ fields [47]. Now, it is of paramount importance that the remaining PGRs be conserved to sustain the agricultural production system in this era of climate change, global environmental problems, and booming population growth [48].

Since the 16th century, more than 80,000 plant species have been collected and preserved in about 3400 gardens across the world [49]. The main objective of this effort is to conserve the PGR diversity and wild species of crop plants to be used in breeding programs. In the mid-20th century, PGRs for food and agriculture (PGRFA) were preserved ex situ in specialized repositories, often termed gene banks. These gene banks are focused on inter-and intra-specific crop diversity. Presently, more than 17,000 regional, national, and international institutions are dealing with the conservation and sustainable use of PGRFA [49]. Additionally, 711 gene banks and 16 regional/international institutions/centers are spread over 90 countries, conserving more than 5.4 million accessions from over 7051 genera. The focus is to conserve the crop species, including crops’ wild relatives, landraces, modern cultivars, genetic stock, and breeding materials [50]. However, various international treaties have been implemented in harmony with the CBD for conservation, sustainable utilization, equity in benefit-sharing, and the safe handling of genetic resources.

### 5.1. International Treaty on Plant Genetic Resources for Food and Agriculture

The International Treaty on Plant Genetic Resources for Food and Agriculture (ITPGRFA) came into force in 2004. ITPGRFA works in harmony with the CBD for sustainable agriculture and food security. The objective of the treaty is the conservation and sustainable use of plant genetic resources for food and agriculture and the fair and equitable sharing of the benefits arising from their use. The conservation and sustainable use of PGRFA are essential to achieving sustainable agriculture and food security, for present and future generations, and are indispensable for crop genetic improvement in adapting to unpredictable environmental changes and human needs.

### 5.2. Nagoya Protocol

The Nagoya Protocol, which came into force in 2014, aims to access genetic resources and encourage the fair and equitable sharing of benefits arising from their utilization. The Nagoya Protocol helps in ensuring benefit-sharing, creates incentives to conserve and sustainably use genetic resources, and therefore, enhances the contribution of biodiversity to development and human well-being.

### 5.3. Svalbard Global Seed Vault

The Svalbard Global Seed Vault situated in Norway safeguards duplicate seed varieties from almost every country in the world. The Seed Vault is owned and run by the Ministry of Agriculture and Food on behalf of the Kingdom of Norway and is established as a service to the world community. The Global Crop Diversity Trust provides support for the ongoing operations of the Seed Vault, as well as funding for the preparation and shipment of seeds from developing countries to the facility. The Nordic Genetic Resource Center (NordGen) operates the facility and maintains a public online database of samples stored in the seed vault. It provides insurance against both incremental and catastrophic loss of crop diversity held in traditional genebanks around the world. The Seed Vault offers long-term protection for one of the most important natural resources on Earth. The main purpose is to backup genebank collections to secure the foundation of our future food supply.

### 5.4. The Cartagena Protocol on Biosafety

The Cartagena Protocol on Biosafety’s goal is to provide safety in the handling of genetic resources, particularly genetically modified organisms. It is an international agreement that aims to ensure the safe handling, transport, and use of living-modified organisms (LMOs) resulting from modern biotechnology that may have adverse effects on biological diversity, while also taking into account risks to human health.

**Figure 2:**
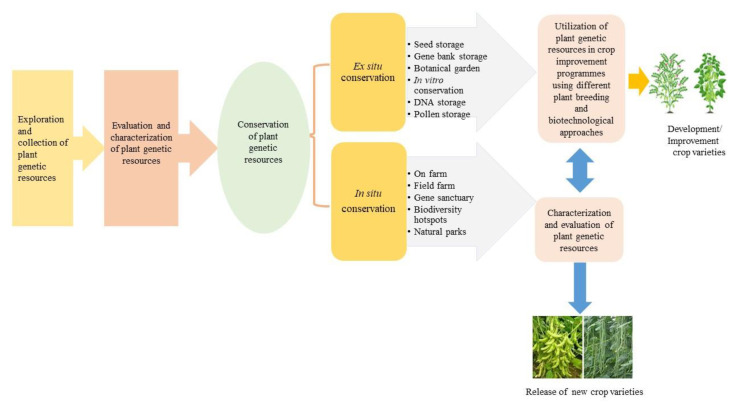
Different strategies used for in situ and ex situ conservation of plant genetic resources.

The ever-increasing demand resulting from the explosive growth rate of the human population worldwide, and global warming, have forced world communities to think about the sustainable use of PGRs. The conservation of PGRs, including landraces, obsolete varieties, breeding material, wild species, and their wild relatives, is of utmost importance to secure future food security [44]. The vanishing of valuable genetic resources invoked the world’s communities to explore, collect, and preserve PGRs and maintain genetic diversity, as well as sign the CBD event in Rio de Janeiro in 1992. The importance of PGRs and biodiversity conservation was the main international issue discussed at the convention [44]. The CBD was organized with three main objectives: (i) the conservation of biodiversity, (ii) the sustainable use of its components, and (iii) the equitable sharing of benefits arising from the use of genetic resources. There is an urgent need to conserve genetic resources for the welfare of human beings and future food security, and to avoid the loss of valuable novel genes. Effective policies should be implemented to evade the extinction of valuable PGRs. There are various methods to conserve biodiversity, such as (i) in situ conservation, (ii) ex situ conservation, and (iii) biotechnological strategies/approaches (Figure 2). The genetic diversity in PGRs, in situ or on farms/fields, is creating awareness in society at large about the importance of agrobiodiversity. In situ and ex situ conservation are complementary strategies to prevent the mass erosion of genetic resources. The utilization of crop genetic diversity is necessary for the development and release of new, well-adapted, and improved varieties for global food security.

### 5.5. In-Situ Conservation

In in situ conservation, genetic resources are conserved in their natural habitat, and the species are maintained in their original place. The plant species are conserved where they are found and are maintained in their original location [51]. In in situ conservation, the process of evolution is allowed to occur naturally with minimum interventions from humans. In this system, many wild plant species are conserved, especially forest and wild fruit crops. In situ conservation permits the plant species to evolve so that genetic diversity can be fostered. This process works via two methods: (i) farm/field conservation and (ii) genetic reserve conservation. Though both are concerned with the conservation and maintenance of diversity of genetic resources, on-farm conservation concerns traditional crop varieties or farming systems, while the latter deals with wild species in natural habitats [4,11]. In genetic reserve conservation, the area is defined by a location where genetic diversity has to be maintained through active and long-term conservation, such as a forest reserve area. In on-farm conservation, locally developed landraces are sustainably managed. Additionally, farmers conserve wild relatives and weedy forms within the existing farming system. Farmers select desirable plants for further cultivation; hence, a continuous process of evolution takes place. The in situ method of conservation allows the open pollination of different genotypes, and the resultant population of different genotypes possesses several alleles. However, to avoid natural calamities and the adverse effects of climate change, both in situ and ex situ conservation should be adopted complementarily [4,11].

### 5.6. Ex-Situ Conservation

Ex situ conservation is the conservation of different genetic resources outside their natural habitat. It involves the conservation of seed gene banks, plant tissue culture, cryopreservation, greenhouses, etc. It is the process of conserving endangered and overexploited genetic resources outside their natural habitat, which otherwise may experience habitat destruction and degradation, and every PGR may go extinct. Therefore, ex situ conservation is an alternate method of conserving valuable genetic resources [52]. In this method, PGRs are saved from extinction that would result from natural calamities, human interference, climate change, over-exploitation, and overutilization. The collected genetic resources should be well evaluated and characterized to avoid duplication, documented, and conserved under artificial conditions to be safe from external threats [4]. Among the various techniques of ex situ conservation, the seed storage technique is the most convenient and easiest for the long-term storage of seeds [4,11]. Orthodox seeds of food crops are used for storage as they can tolerate low temperatures and intense dehydration. In ex situ conservation, about 45% of the stored accessions are seed materials of cereal crops such as rice, wheat, maize, oat, triticale, rye, sorghum, and barley, followed by food legumes (15%), forages (9%), and vegetables (7%) [46,49].

Generally, the conservation of collected seeds is carried out in two ways: base collection and active collection. Base collection is the collection and maintenance of seed samples for long-term conservation. In this case, the seed samples are stored for the maximum time of seed viability at −18 to −20 °C [53]. In the base collection method, the moisture content of the seed to be stored should be between 3% and 7%, depending on the species. In the active collection method, the seed samples are stored for immediate use. Seed samples are stored for 10–20 years and should have at least 65% viability. In the active collection method, the moisture content varies from species to species, i.e., between 7% and 11% for seeds with good storability and between 3% and 8% for seeds with poor storability. It also depends on the temperature under which the seed samples are stored [54]. However, depending on the storage duration, these are categorized into three basic types: (i) long-term storage: when the seed samples are stored in facilities of base collection and are maintained at −18 to −20 °C; (ii) medium-term storage: when the period of storage is not more than 5 years, and seed samples are stored at a temperature between 0 °C and 10 °C with a relative humidity of 20–30%; and (iii) short-term storage: where the seed samples are stored for between 1 year and 18 months. For the latter, the temperature ranges between 20 °C and 22 °C, and the relative humidity should be 45–50%, where the seed can be stored for up to two years without losing its viability [54]. For long-term ex situ conservation, seed storage is the most low-cost and widely adopted storage method. It involves the desiccation of seeds and even storage in low-temperature conditions. However, the recalcitrant seeds and vegetatively propagated plant species do not survive under low temperatures like orthodox seeds. This method is significant for the conservation of forest and tree species. Even novel PGRs can be conserved in the home garden for future use in breeding programs. The ex situ conservation method enables the conservation of novel genes/alleles and ensures their sustainable use in crop improvement programs. The ex situ conservation of PGRs was started in the mid-20th century to slow the rapid loss of biodiversity with modern high-yielding crop varieties. The farmers replaced their traditional cultivars with improved ones [53]. This method is also helpful in the protection and conservation of wild relatives [55,56]. Ex situ conservation methods have been used for conserving important PGRs in several institutes [3,53] (Table 1).

### 5.7. Biotechnological Approaches

Plant biotechnology tools provide new opportunities for the conservation of genetic resources using various in vitro culture techniques. Various biotechnological tools, such as cell and tissue culture and other micropropagation techniques, have greatly contributed to the storage and transportation of PGRs [57]. Cell and tissue culture techniques are in use for the mass multiplication and production of PGRs in a short time for further conservation and transportation under aseptic conditions. Apart from this, next-generation sequencing (NGS), cell fusion techniques, recombinant technologies, proteomic structural biology, protein engineering, and genome editing techniques have opened new avenues and options for conserving genetic resources with increased precision. These technologies help in the conservation of rare and endangered species, ornamental species, forest species, medicinal species, and other vegetatively propagating plant materials [51]. When the biological material of PGRs (such as seeds or organs) cannot be propagated and stored using traditional methods, biotechnological tools, such as in vitro culture, cryopreservation, and molecular biology, can be used. Sometimes, reproductive barrier problems existing in some endangered and rare plant species can be solved via biotechnological interventions [58]. The following subsections highlight the main biotechnological techniques used for PGR conservation, which are not possible under normal storage systems. These techniques also help in the conservation of elite and pathogen-free plants in the short-, medium- and long-term.

#### 5.7.1. In Vitro Propagation

In vitro gene banks are where PGRs are stored in an artificial nutrient medium. This is an alternative method to conserving the vegetative propagated plant genetic material [59,60]. The in vitro conservation method is well recognized by global agencies such as the International Board for Plant Genetic Resources (IBPGR) for safe transportation under regulated phytosanitary control. The main advantages of this technique are insect- and disease-free material, mass multiplication, no genetic erosion, reduced space and labor requirements, and less time taken to obtain a new plant. This technique helps to scale up the production of quality planting material throughout the year. In in vitro methods, the callus is produced from explants such as seeds, leaves, tubers, shoots, and nodes, from which a whole plant is regenerated. In in vitro techniques, an effective conservation method is required once cultures are established and the plant genetic material is multiplied sufficiently. This can be achieved by regularly subculturing the plants onto fresh media. However, there is a risk that subculturing may lead to microbial contamination and chances of somaclonal variations.

The successful production and propagation of genetically stable plants from cultures are prerequisites for in vitro conservation. Shoots are used for slow-growth storage to avoid somaclonal variations. This slow-growth storage is optimal for the medium-term conservation of PGRs [61,62,63]. In this method, the targeted germplasm is stored under plant tissue culture conditions and maintained on nutrient gels for 1 to 15 years with intermittent subculturing. Several techniques are optimized to slow the rate of growth, such as low-intensity light with lower temperatures or a reduced photoperiod. Sometimes, the slow growth of cold-tolerant plant species is maintained by employing a temperature range of 0–5 °C, and for tropical plant species, a temperature range of 15–20 °C. The use of growth retardants in culture media and cutting the supply of oxygen is carried out at different levels to slow down the growth of plantlets [64].

#### 5.7.2. Cryopreservation

The cryopreservation technique involves the storage of biological plant tissue for conservation at ultra-low temperatures (−196 °C), mostly using liquid nitrogen. In cryopreservation, the plant species can be stored for a long period as all the activities, such as cellular metabolism and cell division in recalcitrant seeds and vegetatively propagated plant material, stop. In this approach, no sub-culturing is required, and the chances of somaclonal variations are also reduced [65,66]. The cryopreservation technique ensures cost-effective and safe long-term conservation of plant species; a wide range of plant species can be stored using this technique. In cryopreservation, a cryotherapy technique is also applied to eradicate systemic plant pathogens. In this technique, only meristem cultures or shoot apices are recommended because of their high rate of viability following an extended storage time and because they are virus-free plant materials [67]. In the cryopreservation technique, the first step is to remove all freezable water content from tissues using osmotic dehydration or a physical approach, followed by ultra-rapid freezing [68]. The freezable water content can be removed using freeze-induced dehydration and vitrification methods. In vitrification, crystalized ice formation is avoided, and the liquid phase is directly converted into an amorphous phase [69].

#### 5.7.3. DNA Banks

Advances in molecular biology have made the conservation of endangered and rare species complementary. Genetic resource conservation through DNA is a cost-effective form of conserving PGRs. In biodiversity, many species are difficult to conserve and are at the stage of extinction. DNA storage may be one of the best alternatives to conserve the genetic diversity of these resources, which could possess novel genes/alleles that could aid in future food security. In a gene bank, genomic fragments consisting of individual genes or entire genotypes are conserved in a gene library or a library of DNA samples. Genetic information can be stored in the form of DNA, RNA, and cDNA. These libraries are the primary source of important germplasm for future scientific research worldwide. DNA conservation is an alternative method of conserving PGRs, where the genetic materials are difficult to conserve or threatened because of wild populations or climate change [70]. The genetic material, in the form of DNA, can be stored at −20 °C for up to 2 years for short- and mid-durations. However, for long-term storage, the genetic material can be stored at −70 °C with the help of liquid nitrogen. For the preservation of DNA, there are some DNA banks, such as the Australian Plant DNA Bank of Southern Cross University, the Royal Botanic Garden (UK), the Leslie Hill Molecular Systematics Laboratory, and the US Missouri Botanical Garden. Among these, The Royal Botanic Garden (UK) is the oldest and the most comprehensive DNA bank, encompassing more than 20,000 DNA samples of all plant families. Like other techniques of PGR conservation, DNA conservation can neither constitute the whole plant from conserved DNA nor recover the original genotypes. In these techniques, conserved DNA in the bank is first artificially introduced into the somatic cells, and then, the whole plant is regenerated using in vitro tissue culture techniques [51].

#### 5.7.4. Digital Sequence Information

Digitized molecular data are vital to numerous aspects of scientific research and genetic resource use. Substantial advances in DNA sequencing over the last decades hold great potential to enhance food security and the sustainable use of global biodiversity, benefiting the world’s poorest people. Digital Sequence Information (DSI) plays a crucial role in catalyzing research applications that can contribute to international societal and biodiversity conservation targets. There are concerns over access to genetic resources and the absence of benefit sharing by provider countries. Open access to DSI might exacerbate this, which is leading to increasing policy interventions and restricted access to genetic resources and DSI. However, benefit sharing related to DSI is difficult to identify and hindered by the lack of clear international governance and legislation, which, in turn, has led to a reluctance to make DSI publicly and freely available.

## 6. Utilization of Plant Genetic Resources in Crop Improvement

Before modern cultivated crop varieties, landraces had more genetic diversity. Modern varieties are developed for specific traits, such as high yield, disease resistance, insect pest resistance, stress tolerance, and the improvement of nutritional characteristics. The plant breeders select diverse parents from PGRs in crossing programs to develop new crop varieties [71]. New crop varieties take at least 8–11 years to develop and may last for 5–6 years under cultivation. However, these varieties can be improved further by incorporating novel genes/alleles from wild relatives or wild species. The wild relatives and landraces are rich sources of novel genes resistant to biotic and abiotic stresses, and these are easily crossable with the cultivated crop varieties [72]. PGRs can be used in breeding programs in four ways: (i) the development of pre-breeding materials to be used in traditional breeding methods, (ii) the development of genetic stock as a source of resistance to various biotic and abiotic stresses and quality traits, (iii) the characterization and identification of PGRs for male sterility for the development of hybrids [73], and (iv) the development of modern cultivars by transferring the gene of interest from different genetic resources to popular crop varieties. PGRs are also used to increase genetic variation in the breeding population, incorporate genes to reduce the bottlenecks of the varieties, and develop hybrids, i.e., composites or synthetics.

### Genomic Tools for Efficient Use of Plant Genetic Resources

With the advent of modern biotechnological techniques, the efficiency of plant breeders has significantly increased in the development and improvement of crop varieties. Next-generation sequencing (NGS), high-throughput sequencing (HTS), and high-throughput phenotypic (HTP) techniques enable more efficient use of PGRs. Among the various available techniques, DNA-based techniques are more reliable and widely used in crop improvement programs. Unlike other markers, molecular markers are not influenced by the environmental changes and developmental stages of plants [74]. Molecular markers—such as restriction fragment length polymorphisms (RFLPs), random amplified polymorphic DNA (RAPD), amplified fragment length polymorphisms (AFLPs), inter-simple sequence repeats (ISSRs), diversity array technology (DArT), simple sequence repeats (SSRs), single nucleotide polymorphisms (SNPs), etc.—are widely used in the molecular characterization of genetic diversity present within and between plant populations. Among the various molecular markers, SSR markers are widely used to characterize genotypes [22,75]. DNA-based markers are more efficient in the evaluation of the genetic diversity of endangered and rare species. The main advantages of molecular markers are that any small sample of plant material can be used for genetic diversity analysis. Molecular markers have been widely used to study genetic diversity, and different core collections of PGR accessions have been developed in crops such as rice [66,76,77], wheat [78], mungbean [79], soybean [80], common bean [81,82], pigeon pea [83], chilli [84], potato [85], carrot [86], tomato [87], oil palm [88], cotton [89], mulberry [90], barnyard [91], legume crops [92], and other vegetable and horticulture crops [93].

With the advances in high-throughput sequencing techniques, SNP markers are preferred for use in crop improvement programs. Various QTLs have been identified by developing a biparental population using PGR populations [94,95,96]. Besides the molecular characterization of the germplasm of PGRs for genetic diversity studies, molecular markers are widely used in plant breeding approaches, such as (i) the molecular marker-assisted testing of breeding materials for parental selection, assessing the level of genetic diversity, studying heterosis, the identification of genomic regions under selection, and the assessment of cultivar purity and cultivar identity [35,97,98,99,100,101,102]; (ii) marker-assisted recurrent selection (MARS) [103]; (iii) marker-assisted backcross breeding (MABB) [104]; and (iv) marker-assisted gene pyramiding [105] and genomic selection for complex traits [106]. Important crops have had biotic and abiotic resistance and quality traits introgressed through MAS and MABC/MABB approaches [66,75,92] (Table 2).

Biotechnological tools have been efficiently used for the improvement of susceptible crop varieties. However, for the sustainable utilization of genetic resources, advanced techniques, such as NGS, HPG, and HTP, should be used to develop new crop varieties to ensure food security in the near future.

## 7. Conclusions

To meet the ever-increasing demand for food production, crop diversification, climate-resilient farming, etc., PGRs should be used for sustainability for future food security. However, the efficient use of PGRs can help to meet these needs, and one of the major challenges in the PGR community is to improve access to PGR collections by increasing the amount of information available about collections, through conservation, by participating in pre-breeding activities, etc. A great challenge in the PGR community is the increased demand for PGRs in the wider farming and breeding community. Maintaining genetic diversity, the conservation of PGRs, and sustainable utilization should be the priority of national and international communities. The proper monitoring of genetic erosion and genetic diversity vulnerability is crucial to protecting rare and endangered plant species. As no single technique of conservation is perfect, there is a need to practice in situ and ex situ conservation complementarily. Technical as well as financial support should be provided to farmers and local people for the proper conservation of plant genetic resources. For the management and sustainable use of PGRs, the capacities of local communities, indigenous people, farmers, breeders, extension workers, and other stakeholders, including entrepreneurs and small-scale enterprises, should be strengthened. The proper evaluation, characterization, and documentation of endemic plant species and their exact habitats should be prioritized. More frameworks and policies should be implemented for the sustainable conservation of landraces and their wild relatives. Biotechnological tools should be used for the characterization of plant genetic resources, conservation, and their utilization in breeding programs. Allele/gene mining for important traits in wild species and wild relatives of crops should be given more importance. The effective utilization of plant genetic resources would contribute to solving constraints that limit crop productivity. High-throughput genotypic and phenotypic techniques should be used for the sustainable utilization of genetic resources for future food security.

## Figures and Tables

**Table 1:** Important research institutes conserving and maintaining PGRs.

S. No.	International Research Institute	Mandate/Crops
1.	International Rice Research Institute (IRRI), Los Banos, Philippines	Rice
2.	Centre International de-Mejoramients de Maize (CIMMYT), Trigo, El Baton, Mexico	Maize and wheat (triticale, barely, sorghum)
3.	Center International de-agricultural Tropical (CIAT), Palmira, Columbia	Cassava and beans (also maize and rice), in collaboration with CIMMYT and IRRI
4.	International Institute of Tropical Agriculture (IITA), Ibadan, Nigeria	Grain legumes, roots and tubers, farming systems, cassava, banana, yam
5.	Centre International de la Papa (CIP), Lima. Peru	Potato, Andean root, and tubers
6.	International Crops Research Institute, for Semi-Arid Tropics (ICRISAT), Hyderabad, India	Sorghum, groundnut, pearl millet, Bengal gram, red gram
7.	West African Rice Development Association (WARDA), Monrovia, Liberia	Regional cooperative rice research in collaboration with IITA and IRRI
8.	International Plant Genetic Research Institute (IPGRI), Rome Italy	Genetic conservation
9.	National Bureau of Plant Genetic Resources, New Delhi, India	Fruits, tubers, medicinal and aromatic crops, spices, bulbous crops
10.	The Asian Vegetable Research and Development Center (AVRDC), Taiwan	Tomato, onion, peppers, Chinese cabbage
11.	International Center for Tropical Agriculture (CIAT) Columbia	Cassava
12.	The New Zealand Institute for Plant and Food Research Limited, New Zealand	Kiwifruit (*Actinidia* spp.)
13.	Svalbard Global Seed Vault, Norway	All crops from different countries

**Table 2:** Improvement of various crops using biotechnological tools.

Crop	Molecular Breeding Approaches	Trait(s) Improved	Reference
Rice	MABB	Bacterial blight resistance	[107]
	MABB	Semi-dwarf and bacterial blight resistance	[104,108]
	MABB	Blast resistance	[102]
Wheat	MABB	Stripe rust resistance	[109]
	MABB	Stem rust resistance	[110,111]
	MAS	Leaf rust resistance	[112]
Maize	MABB	Quality improvement	[113]
Cowpea	MABC	Mosaic virus (CpMV) resistance	[114]
Common bean	MABB	Improved drought adaptation	[115]
	MABC	Anthracnose resistance	[116]
Soybean	MAS and MABC	Several soybean cyst nematodes and multiple disease-resistant genotypes	[117]
	MABB	Powdery mildew diseases resistance	[118]
	MABC	Soybean mosaic virus (SMV) resistance	[119]
	MABC	Free Kunitz trypsin inhibitor	[120]
	MABC	Elimination of lipoxygenase-2,	[121]
Peanut	MABC	Introgression lines showing higher yield and increased rust resistance	[122]
	MABC	Resistance to nematode	[123,124]
	MABC	Enhanced oleic acid	[125,126]
Chickpea	MABC	Resistance to fusarium wilt	[127]
	MABC	Resistance to Ascochyta blight	[127]
	MABC	Drought tolerance	[128]
	MABC	Elimination of lipoxygenase-2,	[121]

MAS—marker-assisted selection; MABB—marker-assisted backcross breeding; MABC—marker-assisted backcross.

## Data Availability

No new data were created or analyzed in this study. Data sharing is not applicable to this article.
